# Spatial Heterogeneity of Habitat Suitability for Rift Valley Fever Occurrence in Tanzania: An Ecological Niche Modelling Approach

**DOI:** 10.1371/journal.pntd.0005002

**Published:** 2016-09-21

**Authors:** Calvin Sindato, Kim B. Stevens, Esron D. Karimuribo, Leonard E. G. Mboera, Janusz T. Paweska, Dirk U. Pfeiffer

**Affiliations:** 1 National Institute for Medical Research, Tabora, Tanzania; 2 Department of Veterinary Medicine and Public Health, Sokoine University of Agriculture, Morogoro, Tanzania; 3 Southern African Centre for Infectious Disease Surveillance, Morogoro, Tanzania; 4 Veterinary Epidemiology, Economics & Public Health Group, Department of Production & Population Health, Royal Veterinary College, London, United Kingdom; 5 National Institute for Medical Research, Dar es Salaam, Tanzania; 6 Center for Emerging and Zoonotic Diseases, National Institute for Communicable Diseases, of the National Health Laboratory Service, Sandringham, South Africa; 7 School of Pathology, Faculty of Health Sciences, University of the Witwatersrand, Johannesburg, South Africa; University of California, Davis, UNITED STATES

## Abstract

**Background:**

Despite the long history of Rift Valley fever (RVF) in Tanzania, extent of its suitable habitat in the country remains unclear. In this study we investigated potential effects of temperature, precipitation, elevation, soil type, livestock density, rainfall pattern, proximity to wild animals, protected areas and forest on the habitat suitability for RVF occurrence in Tanzania.

**Materials and Methods:**

Presence-only records of 193 RVF outbreak locations from 1930 to 2007 together with potential predictor variables were used to model and map the suitable habitats for RVF occurrence using ecological niche modelling. Ground-truthing of the model outputs was conducted by comparing the levels of RVF virus specific antibodies in cattle, sheep and goats sampled from locations in Tanzania that presented different predicted habitat suitability values.

**Principal Findings:**

Habitat suitability values for RVF occurrence were higher in the northern and central-eastern regions of Tanzania than the rest of the regions in the country. Soil type and precipitation of the wettest quarter contributed equally to habitat suitability (32.4% each), followed by livestock density (25.9%) and rainfall pattern (9.3%). Ground-truthing of model outputs revealed that the odds of an animal being seropositive for RVFV when sampled from areas predicted to be most suitable for RVF occurrence were twice the odds of an animal sampled from areas least suitable for RVF occurrence (95% CI: 1.43, 2.76, p < 0.001).

**Conclusion/Significance:**

The regions in the northern and central-eastern Tanzania were more suitable for RVF occurrence than the rest of the regions in the country. The modelled suitable habitat is characterised by impermeable soils, moderate precipitation in the wettest quarter, high livestock density and a bimodal rainfall pattern. The findings of this study should provide guidance for the design of appropriate RVF surveillance, prevention and control strategies which target areas with these characteristics.

## Author Summary

Rift Valley fever is a viral disease that is transmitted to livestock and humans by mosquitoes. Humans get infected mainly through direct contact with blood or aborted materials from infected animals. In Tanzania, a total of 10 RVF epidemics have been reported from 1930 to 2007. Despite the long history of RVF in Tanzania, the extent of its suitable habitat remains unclear. As a result, disease prevention measures such as vaccination of livestock are implemented without informed risk-based resource-allocation decisions. This study was therefore carried out to identify the locations in Tanzania where RVF is more or less likely to occur using an ecological niche modelling (ENM) method. Data from 193RVF outbreak locations were used together with precipitation, elevation, soil type, livestock density, rainfall pattern, proximity to wild animal protected areas as well as to forest as inputs for ENM. Our results show that locations at most risk for RVF occurrence were the northern and central-eastern Tanzania. Areas at highest risk for RVF occurrence are characterised by soils with low water permeability, high amounts of rainfall, high livestock density and two rainy seasons in a year. The findings of this study provide guidance in the design of appropriate RVF surveillance, prevention and control strategies that can be implemented cost-effectively by targeting the areas at most risk.

## Introduction

Rift Valley fever (RVF) is a mosquito-borne zoonotic disease of major public health and economic concern occurring mainly in Africa [1–6] and the Arabian Peninsula [7, 8]. The potential for further geographical spread of RVF to other areas of the world has been suggested [9–11]. The disease is caused by the RVF virus (RVFV) of the genus *Phlebovirus* and family *Bunyaviridae* [12, 13] and affects both humans and livestock. In this study, RVF outbreak was defined as occurrence in a specific location of laboratory-confirmed RVF cases affecting domestic ruminants. A RVF outbreak wave (epidemic) referred to sequential reports of the outbreaks at various locations within Tanzania from date of onset of the first outbreak during a particular time period of the year until outbreaks were no longer reported in the country. Tanzania has a long history of RVF outbreaks, and it is not known how RVFV was introduced to the country. Between 1930 and 2007, a total of 10 RVF outbreak waves have been reported in Tanzania with average inter-epidemic period (IEP) of 8 years [14–17]. There also appears to be spatial heterogeneity in the distribution of RVF. A total of 31/90 (34.4%) districts from 10/14 (71.4%) regions in the eastern Rift Valley ecosystem have reported RVF outbreaks in the past compared with 12/69 (17.4%) districts from 5/11 (45.5%) regions in the western ecosystem [18–20]. The past RVF outbreaks in Tanzania resulted in devastating socio-economic losses including food insecurity and threatened livelihoods. Notably, the last RVF outbreak in Tanzania in 2006/2007 caused high mortality rates in laboratory confirmed cases amongst domestic ruminants (37%, n = 136,570) and humans (46%, n = 309) [14]. Animals lost monetary value by 34% (e.g. price of a bull dropped from US$ 238 to 158), monthly internal market flow dropped by 37% (e.g. 4,251 to 2,679 cattle) and annual external market flow dropped by 54% (e.g. 2,594 to 1,183 cattle) [14]. Additionally, the loss due to death of domestic ruminants was > US$ 6million and the government spent about US$ 4 million in the control of the disease [14].

It is not known why RVF outbreaks have been reported mainly in the eastern Rift Valley ecosystem. However, it should be realized that active and well-structured RVF surveillance has never been conducted throughout the country partly because of financial resources and challenging logistics. The northern Tanzania, particularly Ngorongoro district, in the eastern Rift Valley ecosystem has remained the epicentre of all past RVF outbreaks in the country [16]. As a result, past RVF surveillance and awareness campaign efforts have been concentrated much more in the northern than other areas of the country. We cannot therefore, discount the possibility that sampling or reporting bias may have contributed to over-reporting of RVF outbreaks in the eastern rather than the western Rift Valley ecosystem over time. It is probable that some of the un-sampled locations and locations without reports of RVF outbreaks in the country are also suitable for disease occurrence. Because the disease control resources are generally limited, it is interesting to understand if heterogeneity exists in the habitat suitability for RVF occurrence in the country, as this will inform allocation of disease prevention and control resources proportional to the risk.

A number of scientific methods are available that can be used to generate information on the potential suitable habitat for species and disease occurrence. These include general-purpose statistical methods of temporal and spatial prediction such as generalized linear models (GLM) [18, 19], generalized additive models (GAM) [20, 21] and Bayesian estimation methods [22, 23]. However, such models require both disease presence and absence data and inferences drawn from their outputs are therefore limited to the area covered by the data. Furthermore, these methods frequently fit linear functions between predictor variables and disease data although ecological associations are frequently highly complex and non-linear [24, 25].

Ecological niche models (ENMs) that were originally developed for ecological and conservation purposes are being used increasingly to model the spatial distribution and potential risk of occurrence of a range of diseases and vector species. For example, they have been applied to characterize the habitat suitability for leishmaniasis [26], malaria [27–30], RVF [31, 32], bluetongue [33], anthrax [34], dengue [30], Chagas disease [35], filovirus disease [36], Marburg hemorrhagic fever [37], avian influenza [38], plague [39, 40] and lymphatic filariasis [41]. The main advantage of ENMs, over that of the more traditional regression modelling approaches, such as generalized linear mixed models, is that they require only presence data [39]. These data are used, together with a randomly-generated sample of background data points from the study area (representing the available environment) and a suite of predictor variables, to define the fundamental niche of the species or disease [42, 43]. In addition, as the results of such models can be extrapolated beyond the geographical areas defined by the data points used to calibrate the model, these predictive risk mapping approaches are useful for identifying other areas suitable for occurrence of the disease [42]. These presence-only methods illustrate the likelihood of an organism’s presence or the relative ecological suitability of a spatial unit within the study area [43]. Maximum Entropy (MaxEnt) is one of the presence-only general-purpose niche modelling algorithms, which has been described as efficient to estimate the probability distribution of species and diseases [42–48] and is reported to perform well, even with very small sample sizes [49–50].

In this study, we investigated the potential effect of bioclimatic variables related to temperature and precipitation, elevation, soil type, livestock density, rainfall pattern, proximity to wild animal protected areas and proximity to forest on the spatial habitat suitability for RVF occurrence in Tanzania. We anticipate that generation of evidence-based information on the spatial dimensions of the potential suitable habitat of RVF occurrence and understanding how much the potential predictor variables contribute in delineating these suitable habitats, will inform targeted risk assessment, surveillance and cost-effective-usage of disease control and prevention resources.

## Methods

### Ethics statement

The domestic ruminants (cattle, sheep and goats) RVF disease outbreak data used in this study were extracted from reports of the ministry responsible for livestock development in Tanzania. These data were anonymous, and it was therefore not possible to associate disease data with specific animal or its owner. Serological data from domestic ruminants (cattle, sheep and goats) used for ground-truthing of the ecological niche modelling outputs were from the study that received ethical approval from the Medical Research Coordinating Committee of the National Institute for Medical Research in Tanzania (ethics certificate number NIMR/HQ/R.8a/Vol.IX/1296).

### Study area

**Fig 1:**
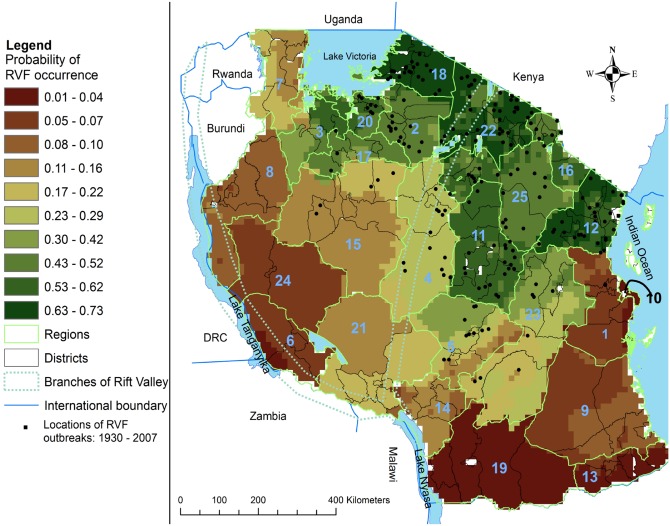
Probability of Rift Valley fever occurrence in Tanzania overlaid with locations of RVF outbreaks in domestic ruminants 1930–2007. Key for regions: 1- Pwani; 2- Simiyu; 3- Geita; 4- Singida; 5- Iringa; 6- Rukwa; 7- Kagera; 8- Kigoma; 9- Lindi; 10- Dar es Salaam; 11- Dodoma; 12- Tanga; 13- Mtwara; 14- Njombe; 15- Tabora; 16- Kilimanjaro; 17- Shinyanga; 18- Mara; 19- Ruvuma; 20- Mwanza; 21- Mbeya; 22- Arusha; 23- Morogoro; 24- Katavi and 25- Manyara.

This study was conducted in Tanzania Mainland, located between longitudes 29 and 41° east and latitudes 1 and 12° south. Tanzania Mainland borders Kenya, Uganda and Lake Victoria in the north, Rwanda, Burundi and the Democratic Republic of the Congo (DRC) in the west. On the south it borders with Zambia, Malawi, Mozambique and Lake Nyasa, and to the east it borders the Indian Ocean (Fig 1). Administratively, Tanzania Mainland has 25 regions with total land areas of 883,343 square kilometres. The ecological characteristics of the country vary widely. The north-eastern regions experience a bimodal rainfall pattern (October—December and March—May) whereas the central, western and southern regions of the country experience a unimodal rainfall pattern (November—May). Pastoralism is mainly concentrated in Arusha and Manyara regions and agro-pastoralism in Tabora, Geita, Shinyanga, Mwanza, Dodoma and Singida regions [51]. The plateau of the northern Tanzania is comprised of relatively higher livestock densities (cattle ≥ 50, goats ≥ 45 and sheep ≥ 14 head per square kilometre) than the rest of the country [51].

### Data sources and preparation

#### Presence point records for RVF outbreaks in Tanzania

The definition of habitat suitability was adapted from Franklin [52], and in this study it refers to the ability of a habitat to support the occurrence of RVF. In this study, we also refer to habitat suitability as probability of occurrence. A total of 303 locational point data of reported RVF outbreaks for domestic ruminants (cattle, sheep and goats) in Tanzania were available through the disease reporting registers from the ministry responsible for livestock development for the period spanning 1930 to 2007, and are presented in Fig 1. Detailed descriptive analysis of these RVF outbreak data is presented in a recent study by Sindato and others [16]. Data on RVF occurrence were available at village and monthly spatial and temporal resolutions, respectively. Villages were identified by specific geo-coordinates. Only one entry record for RVF outbreaks was retained in the dataset for those geo-coordinates with multiple entries for a particular month. Duplicate presence point records for RVF outbreaks were removed using ecological niche modelling tools (ENMTools) software version 1.4.3 [53] leaving a total of 193 RVF outbreak points, which were used to calibrate the ecological niche model, together with 10,000 background points randomly generated by the MaxEnt software version 3.3.3k [42]. Background data are sampled from the whole study area in order to characterize the environmental conditions existing within it (i.e. both presence and absence grid cells are included in the sampling frame) [54]). In addition, the sampling frame for background data should ideally include the full range of environments in which the species can potentially occur. Constraining the background data sampling frame to exclude specific environmental conditions can unintentionally truncate the species’ niche and thereby lead to incorrect feature selection, and consequently, incorrect estimates of suitable habitat [55]. Thus, when selecting the extent of the region from which the background data are sampled, it has been suggested that this should be limited to areas which are accessible via dispersal [55]. As dispersal of RVF is much dependent on vector dispersal and uncontrolled animal movement in Tanzania, we reasoned that all regions of the study area were potentially accessible to the virus and should therefore be included in the background data sampling frame.

#### Potential predictors of RVF occurrence

Potential predictors for RVF occurrence were identified from the literature [14–17, 56–61] and those that can be mapped included elevation, soil types, livestock density, rainfall pattern, proximity to wild animal (national parks, game reserves and conservation areas) and forest (closed forest and woodland) protected areas, and bioclimatic variables related to temperature and precipitation. The 11 bioclimatic variables related to temperature included annual mean temperature, mean diurnal temperature range, isothermality, temperature seasonality, max temperature of warmest month, min temperature of coldest month, temperature annual range, mean temperature of wettest quarter, mean temperature of driest quarter, mean temperature of warmest quarter and mean temperature of coldest quarter. Eight bioclimatic variables related to precipitation included annual precipitation, precipitation of wettest month, precipitation of driest month, precipitation seasonality, precipitation of wettest quarter, precipitation of driest quarter, precipitation of warmest quarter and precipitation of coldest quarter. These bioclimatic layers (related to temperature and precipitation) were downloaded from the World climate website (http://www.worldclim.org/current) at a resolution of 30 arc-seconds (~1km). Data for livestock (cattle, sheep and goats) density were obtained from the ministry responsible for livestock development in Tanzania (available at regional resolution) based on the national sample census of agriculture conducted in 2007/2008, and is available at http://harvestchoice.org/sites/default/files/downloads/publications/Tanzania_2007-8_Vol_5g.pdf. Data for wild animal and forest protected areas (available mainly at district spatial resolution) were downloaded from http://www.tzgisug.org/wp/spatial-data-sources-for-tanzania. Data on soil type was obtained from the Mlingano Agricultural Research Institute in Tanga (available at regional resolution), Tanzania, and is available at http://www.kilimo.go.tz/agricultural%20maps/Tanzania%20Soil%20Maps/Webbased%20Districts%20Agricultural%20maps/Districts%20Soil/Soils%20of%20Tanzania.pdf. ArcGIS 10.2 (ESRI East Africa) was used for all spatial data manipulations. The spatial analysis tool in ArcGIS 10.2 was used to calculate the Euclidean distance to the feature of interest for the ‘proximity to’ spatial data layers. For modelling purposes, all variable layers were clipped to the extent of the country with a resolution of 1 km^2^.

#### Collinearity analysis

Bioclimatic data contain variables describing patterns in temperature and precipitation derived from a common set of temperature and precipitation data, which have been shown to be highly correlated with each other [62–64]. Including highly correlated variables in the model would make it difficult to determine exactly how each variable influences the occurrence of the species or disease [65, 66]. Therefore, preliminary assessment was made to identify a single optimal temperature or precipitation predictor from the set of 19 bioclimatic variables [67] for inclusion in the model as follows: two ecological niche models with default settings in the MaxEnt software were run—one incorporating only eight precipitation-related variables and the second incorporating only 11 temperature-related variables. The single temperature and precipitation variables which best fit the data were selected using the model area under the curve (AUC). These two predictor variables, mean diurnal temperature range and precipitation of wettest quarter, were carried forward for evaluation in the model together with elevation, soil type, livestock density, rainfall pattern, proximity to wild animal protected areas and proximity to forest. Collinearity between each pairs of these eight predictor variable layers was assessed using Pearson correlation analyses in ENMTools version 1.4.3 [53]. Two predictor variable layers were considered highly correlated at a Pearson correlation coefficient value > 0.70. This threshold value of correlation was set conservatively in view of other studies that have considered predictor variables to be highly correlated at correlation coefficient values > 0.35 [66], > 0.75 [68] and > 0.85 [69]. Based on the potential biological relevance to the occurrence of RVF, only one predictor variable from a set of highly correlated variables was included in our model.

### Modelling habitat suitability for RVF occurrence

Ecological modelling of habitat suitability for RVF occurrence was implemented using the MaxEnt software version 3.3.3k [42]. There has been no systematic surveillance of RVF in Tanzania and therefore, the spatial range of its occurrence was not explicitly known. Prior to conducting our study, we could not differentiate whether more RVF cases were confirmed in the northern Tanzania because those locations were suitable for disease occurrence or rather because they received the largest surveillance efforts. Our presence dataset was therefore considered small and biased because of the fact that most of past surveillance efforts have been conducted in the northern Tanzania. We assumed that the un-sampled locations of the country could be suitable for RVF occurrence. For this reason, the MaxEnt default setting seemed more appropriate because it assumes that the species/disease being modelled is equally likely to be anywhere in the geographical space of the study area [70]. In addition, the regularization multiplier was set to 1 to limit over-fitting of the model and prevent prediction from being inadequately large [48]. Regularization multiplier is a parameter that leads to smoothening of the regression line to minimizing the error function and thus prevents over-fitting of the model. It does so by penalizing the values of the features that tries to closely match the noisy data points resulting to balanced optimal solution to avoid making the model complex. The model containing the optimal combination of predictor variables was run with ten replicates and 500 iterations at a convergence threshold of 0.00001, with cross validation replicate type. The output was set to logistic format, so that the predictions of habitat suitability would assume probability scores between 0 and 1 [42].

### Model performance and selection criteria

To determine which set of predictor variables best fit the data, performance and selection criteria were implemented using the MaxEnt software [42] and MaxEnt extension, ENMTools [53]. A backward stepwise approach was implemented in MaxEnt using the jackknife test of relative contribution of the predictor variables in the model as follows. Eight models were run in MaxEnt, starting with one that included all eight predictor variables. In the process of building the model, the variable which contributed the least was removed from subsequent models until only one variable remained. AUC values were recorded for each model. The raw outputs from MaxEnt were further evaluated using ENMTools [53]. The optimal combination of predictor variables included in the final model was the one that generated the largest AUC and at least one of the smallest of Akaike`s information criterion (AIC), sample-size corrected Akaike`s information criterion (AIC_c_) or Bayesian information criterion (BIC) [71, 72]. The percentage contribution and permutation importance were computed for each predictor variable. The magnitude of change in training AUC represented by the average over the 10 replicate runs was normalized to percentages. The higher the percentage contribution, the more impact that particular variable had on predicting the most suitable habitat for RVF occurrence [53]. In order to assess the training gain of each predictor variable, the jackknife of regularized training gain was produced by running the model in isolation and comparing it to the training gain of the model including all variables. This was used to identify the predictor variable that contributed the most individually to the habitat suitability for RVF occurrence. The response curves describing the probability of RVF occurrence in relation to the different values of each predictor variable were generated using only the variable in question and disregarding all other variables. The contribution of each predictor variable to the final model was assessed using the jackknife procedure based on the AUC, which provides a single measure of model performance [42]. The probability scores (numeric values between 0 and 1) were displayed in ArcGIS 10.2 (ESRI East Africa) to show the locations in Tanzania where RVF is predicted to be more or less likely to occur.

### Ground-truthing of the ecological niche modelling outputs

Ground-truthing of the ecological niche modelling outputs was conducted by comparing the levels of antibodies specific to RVFV in domestic ruminants (sheep, goats and cattle) sampled from locations in Tanzania that presented different predicted habitat suitability values. We assumed that locations with higher proportions of RVFV-seropositive animals represented higher levels of habitat suitability for RVFV activity than locations with low proportions of seropositive animals. The details of sampling process and laboratory analysis of serum samples have been described by Sindato and others [73]. In brief, MaxEnt predictive map of habitat suitability for RVF occurrence (Fig 1) was used as guidance to purposively identify six villages from six districts in the eastern and western Rift Valley ecosystems of Tanzania as described elsewhere (73). The district veterinary officers were consulted in order to identify one district within the region perceived to be at highest risk of RVF occurrence. Criteria used included presence of shallow depressions/locations that are subject to regular flooding, ecological features suitable for mosquito breeding and survival/experience of mosquito swarms during the rainy season, relatively high concentration of domestic ruminants, proximity to forest, rivers, lakes, wildlife and presence of areas with history of RVF occurrence. The district within the region that was identified to have most of these epidemiological characteristics was selected for the study, even if they had never reported RVF outbreaks. Utilizing local veterinary records, only the villages with livestock that have never been vaccinated against RVF were targeted. Based on the above criteria for identifying the six study districts, additional discussions were then held with local veterinary/agricultural staff, community leaders and livestock keepers to identify one village within each district that was perceived to be at highest risk for RVFV activity. The number of villages surveyed was not based on statistical considerations, but rather logistical and financial factors. The selected villages from the eastern Rift Valley ecosystem were Chamae, Malambo and Ninchoka, and all had reported RVF outbreaks in the past. Selected villages from the western Rift Valley ecosystem were Bukirilo, Nyakasimbi and Kajunjumele, and all had never reported RVF outbreaks. Nyakasimbi village is located in Karagwe district in the western Tanzania bordering with Rwanda, and Kajunjumele village is located in Kyela district in the southern highland bordering with Lake Nyasa. Ninchoka and Malambo villages are located in Serengeti and Ngorongoro districts, respectively, in the northern Tanzania bordering with Kenya. Bukirilo and Chamae villages are in Kibondo and Kongwa districts in the western and central Tanzania, respectively.

Within each selected village a two stage random sampling process was used to select the herds and domestic ruminants. In each of the selected villages, 20 herds keeping at least one of the three domestic ruminant species (cattle, sheep and/or goats) were randomly selected from the list of livestock keepers. Within each herd, a maximum of 20 ruminant animals (not more than 20 animals were selected from a herd) born after the last RVF outbreak in 2006/2007 in Tanzania were bled (i.e. 10 cattle, 5 goats and 5 sheep) depending on the herd size and species composition within the herd at the time of sampling. Collected serum samples were tested for the presence of anti-RVFV antibodies using IgM-capture ELISA [74] and inhibition ELISA [75]. The results were interpreted using the cut-off threshold specified by the manufacturer of the test kit. For IgM capture ELISA method: Sheep, goat and bovine sera producing PP values ≥7.9, 9.5 and ≥ 14.3, respectively, were considered to be positive and less than these values as negative [74]. For RVF inhibition ELISA method: Serum samples with PI equal to or greater than 41.9, 41.4 and 38.4 were considered seropositive for RVF inhibition in cattle, goats and sheep, respectively [75]. The data were analysed using logistic regression modelling to investigate the association between various suitability habitat values (potential predictors) and RVFV seropositivity outcomes in domestic ruminants. Based on the limited resources available and logistic factors, the study sites for model ground-truthing were not selected using simple random sampling approach but rather using a purposive sampling approach. When any sampling method other than simple random sampling is used, the survey data analysis method is used to take into account the differences between the design that was used and simple random sampling. This is because the sampling design affects both the calculation of the point estimates and the standard errors of the estimates (e.g. regression coefficients). When non-independent sampling process is not accounted for in the analysis the standard errors will likely be underestimated, possibly leading to results that seem to be statistically significant, when in fact, they are not. The svy command was therefore used in the modelling process using Stata version 12 (Statacorp, College Station, TX, USA) to account for sample survey design effect.

## Results

### Selection of the final model and analysis of variable contributions

Eight predictor variables, namely mean diurnal temperature range, precipitation of wettest quarter, elevation, soil type, livestock density, rainfall pattern, proximity to wild animal protected areas and proximity to forest were initially evaluated in the model. The pair-wise correlation matrix for these predictor variables suggested that there was moderate correlation between mean diurnal temperature range and precipitation of wettest quarter (r = -0.56), precipitation of wettest quarter and livestock density (r =—0.58) and rainfall pattern and livestock density (r = 0.62) (Table 1). Of the eight predictor variables evaluated in the initial model, four—proximity to forest, proximity to wild animal protected areas, elevation and mean diurnal temperature range—were dropped from the model leaving four predictor variables in the final model (Model_4; Table 2). This model, which contained the predictor variables soil type, precipitation of wettest quarter, livestock density and rainfall pattern was selected as the model of best fit based on the highest mean AUC and lowest BIC, as well as one of the lowest values of AIC and AIC_c_. All subsequent results refer to this model. Soil type and precipitation of wettest quarter together accounted for almost two-third (64.8%), while livestock density and rainfall pattern together accounted for just over one-third (35.2%) of the variation in habitat suitability for RVF occurrence (Table 2).

**Table 1:** Pearson correlation coefficient for pairs of predictor variables associated with occurrence of RVF.

Predictor variable	Soil type	Mean diurnal temperature range (oC)	Precipitation of wettest quarter (mm)	Elevation (metre above sea level)	Livestock density (heads per square km)	Proximity to forest (km)	Proximity to protected areas (km)	Rainfall pattern
**Soil type**	1	-0.10	0.20	-0.22	-0.08	0.09	0.01	-0.28
**Mean diurnal temperature range (oC)**		1	**-0.56**	-0.02	0.25	0.09	0.31	-0.18
**Precipitation of wettest quarter (mm)**			1	0.07	**-0.58**	-0.23	-0.07	-0.26
**Elevation (metre above sea level)**				1	-0.01	-0.06	-0.01	0.09
**Livestock density (heads per square km)**					1	0.22	-0.06	**0.62**
**Proximity to forest (km)**						1	-0.02	0.10
**Proximity to protected areas (km)**							1	-0.13
**Rainfall pattern**								1

**Table 2:** Percentage contribution of individual predictor variables in eight ecological niche models describing the spatial distribution of habitat suitability for RVF occurrence in Tanzania. The number in each model (i.e. 1 to 8) indicates the number of predictor variables that model contained.

Predictors	Models							
	8	7	6	5	*4	3	2	1
Livestock density (heads per square km)	23.1	19.4	25.0	21.2	25.9	29.0	57.9	100.0
Soil type	29.0	30.1	27.0	30.2	32.4	30.0	42.1	
Precipitation of wettest quarter (mm)	25.6	34.9	31.0	35.8	32.4	40.9		
Rainfall pattern	7.7	5.3	7.6	7.4	9.3			
Mean diurnal temperature range (degrees Celsius)	8.4	6.7	6.2	5.4				
Elevation (metre above sea level)	4.1	2.1	3.2					
Proximity to protected areas (km)	1.3	1.5						
Proximity to forest (km)	0.8							
AUC	0.772	0.764	0.787	0.799	0.812	0.798	0.797	0.744
AIC	2721	2718	2773	2772	2771	2944	3005	3043
AICc	2740	2731	2782	2776	2772	2946	3007	3044
BIC	2836	2816	2856	2832	2806	2983	3044	3066

*Best fit ecological niche model. Key: AUC: area under the curve, AIC: Akaike’s information criterion, AIC_c:_ sample-size corrected Akaike’s information criterion and BIC: Bayesian information criterion.

### Habitat suitability map for RVF

The habitat suitability of RVF occurrence in domestic ruminants in Tanzania was displayed on continuous probability scores of least to most suitable represented by a brown-green–colour scale (Fig 1). Probability scores were mapped at district level and the grid size was 1km^2^. It is clear from our results that the habitat suitability of RVF occurrence was heterogeneously distributed throughout the country. About one-third (29%) of Tanzania Mainland area (n = 883,343 Km^2^) comprising 10 (40%, n = 25) regions (dark-green shades in the northern and central-eastern areas of the country) represented highest probability scores and were considered most suitable for RVF occurrence. Almost one-fifth (18%) of the land area comprising three (12%, n = 25) regions represented by light-green in the central-southern areas of the country were considered moderately suitable for RVF occurrence. Over half (53%) of the land area comprising 12 regions (48%, n = 25) represented by light- and dark-brown in the western and south-eastern areas of the country were considered least suitable for RVF occurrence. Predictive performance of the model was considered good with mean test AUC of 0.812 and standard deviation of the mean probability of 0.014 for the 10 replicate runs.

### Jackknife of regularized training gain for RVF habitat suitability

**Fig 2:**
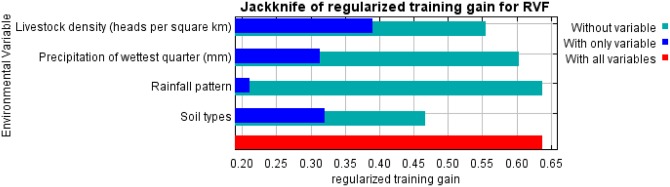
Jackknife of regularized training gain for RVF occurrence.

The results of the jackknife regularized training gain indicated that the predictor variable with the highest gain when used in isolation was livestock density. The predictor variable that decreased the gain the most when it was omitted was soil type. Values shown are averages over 10 replicate runs (Fig 2).

### Jackknife test of variable importance for area under the curve (AUC) of the final model

**Fig 3:**
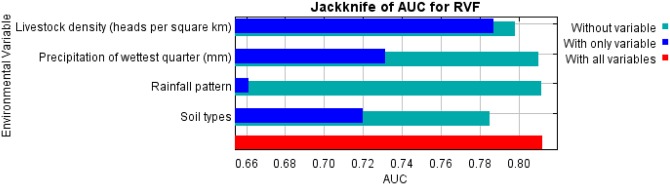
Jackknife test of predictor variables importance on RVF occurrence as determined by the area under the curve (AUC) of the final model.

Jackknife test of variable importance utilizing the AUC showed that livestock density contributed the most to the AUC (longest dark-blue bar), followed by precipitation of the wettest quarter, soil type and rainfall pattern (Fig 3).

### Response graphs for habitat suitability of RVF occurrence

**Fig 4:**
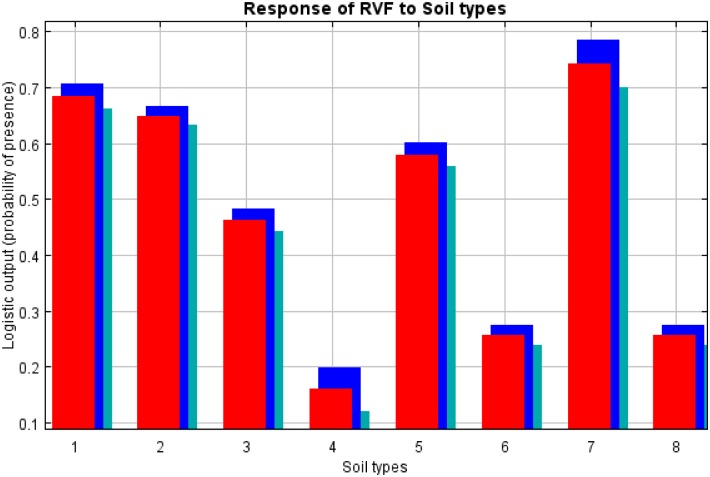
Probability of RVF occurrence in relation to soil types. The red columns present mean response of all 10 replicates, while blue and light green indicate standard deviation of the mean. The key to soil types: 1, chernozems; 2, andosols; 3, acrisols; 4, ferralsols, 5, luvisols; 6, cambisols, 7, planosols and 8, lixisols.

**Fig 5:**
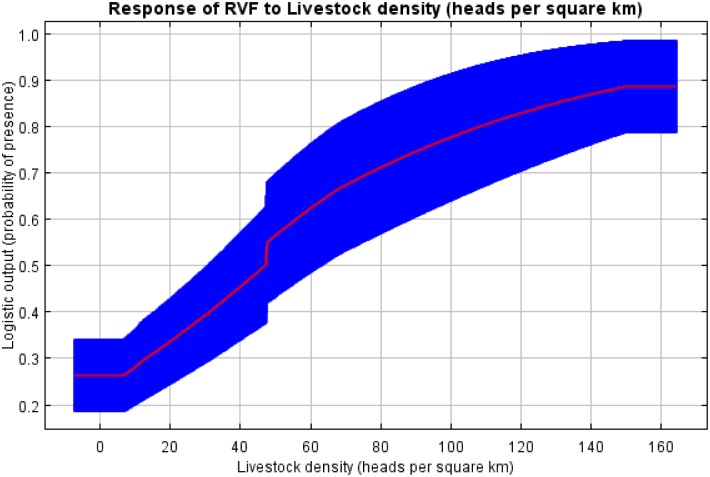
Probability of RVF occurrence in relation to livestock density. The red curved present mean response of all 10 replicates of the model, while blue indicates standard deviation of the mean.

**Fig 6:**
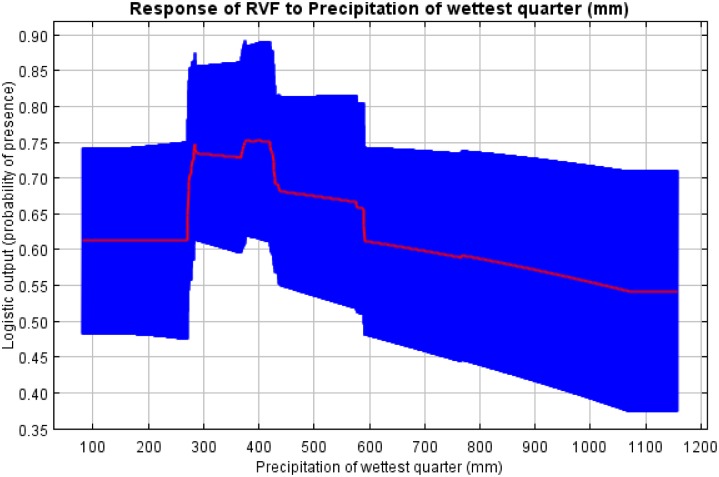
Probability of RVF occurrence in relation to precipitation of wettest quarter. The red curved present mean response of all 10 replicates of the model, while blue indicates standard deviation of the mean.

The response graphs for the final model showed that probability scores were highest in areas with impermeable soils (planosols followed by chernozems, andosols, luvisols and acrisols), while the lowest probability scores were observed in locations with permeable soils (ferralsols, cambisols and lixisols) (Fig 4). The areas that experienced a bimodal pattern of rainfall had much higher probability of RVF occurrence than those that experienced a unimodal rainfall pattern. Probability of RVF occurrence was very low (around 0.26) at minimum values livestock density of < 8 heads/km^2^. It then followed a sigmoidal pattern with an initial increase in probability occurring between 8 and 46 heads/km^2^, a rapid increase between 46 and 48 heads/km^2^and after 150 heads/km^2^ the probability of RVF occurrence remained constant (Fig 5). Probability of RVF occurrence was around 0.62 at the precipitation of the wettest quarter of < 275mm. A sharp increase in the probability of RVF occurrence occurred with the precipitation of the wettest quarter between 275 and 290 mm (Fig 6). The highest probability of RVF occurrence in relation to precipitation of the wettest quarter was 0.76 that occurred between 375 and 425 mm. Then there was a sharp rate of decline in the probability between 425 and 430 mm, slower rate of decline between 430 and 590 mm and a further sharp decline to a probability of 0.60 between 590 and 595 mm. Thereafter there was a further slower rate of decline in the probability to < 0.55 at around 1,075mm (Fig 6).

### Ground-truthing of ecological modelling outputs

According to our ecological niche modelling algorithm; Ninchoka, Malambo and Chamae villages are located in the northern and central areas of the country considered most suitable for RVF occurrence while Kajunjumele, Nyakasimbi and Bukirilo villages are in the western and southern areas of the country considered least suitable areas. A total of 1,435 domestic ruminants from 121 herds (61 herds from western and 60 herds from eastern Rift Valley ecosystem) in these six villages were tested for antibodies against RVFV. About an equal proportion of tested serum samples were collected in livestock from the villages in the districts within the eastern (51.9%) and western (48.2%) ecosystems of the Rift Valley. The number of serum samples from each study village was: Malambo; 243 (16.9%), Ninchoka; 257 (17.9%), Chamae; 244 (17.0%), Nyakasimbi; 233 (16.3%), Bukirilo; 233 (16.3%) and Kajunjumele; 225 (15.7%). Ground-truthing of model outputs revealed a significant variation in the odds of RVFV seropositivity in livestock sampled from locations with different suitability habitat values for RVF occurrence. The odds of an animal sampled from the most suitable location being seropositive for RVFV were two times higher than the odds of an animal sampled from least suitable areas (OR = 2.0, 95% CI: 1.43, 2.76, p < 0.001).

## Discussion

Rift Valley fever is becoming increasingly important owing to its socio-economic and public health consequences. Despite the long history of RVF in Tanzania, the level of disease risk in various locations of the country remains unclear. As a result, disease prevention measures such as vaccination of livestock are implemented without informed risk-based resource-allocation decisions. To be cost-effective, allocation of disease prevention and control resources should be proportional to the risk of RVF occurrence. The findings of this study provide valuable information on the spatial suitability habitat for RVF occurrence in Tanzania, thus greatly assist informed risk-based surveillance, prevention and control activities. Based on the findings of this study, it is credible to suggest that an appropriate RVF intervention strategy in Tanzania should consider implementing disease prevention activities, including pre-emptive vaccination of livestock, by targeting the areas identified to be most suitable for disease occurrence prior the predicted times of high environmental risk. Regular surveillance activities for RVF activity should consider conducting representative sampling of the areas in the country with various habitat suitability values. For surveillance purposes all the suitability classes should be represented in the sample to monitor transmission dynamic of RVF. This is because subsequent RVF outbreaks have expanded to involve new foci in the country over time [16]. This suggests that areas that are currently considered to be at low risk may in future be at high risk because of factors such as uncontrolled animal movements and weather variability over time. To enhance early detection, sentinel surveillance should be conducted in the areas considered to be at most risk for RVF occurrence.

Our findings suggest that used collectively, four predictor variables (livestock density, precipitation of the wettest quarter, soil type and rainfall pattern) in the model resulted in the best model fit. The resulting habitat suitability map of our model suggests that the northern and central-eastern Tanzania has higher values of suitable habitat of RVF occurrence than the rest of the country. The locations in Tanzania which are considered most suitable for RVF occurrence are characterised by bimodal pattern of rainfall, higher livestock density and predominantly impermeable soils i.e. soils that do not easily allow water to filter through. Contrary, the locations in the country which are considered least-moderately suitable for RVF occurrence are characterised by unimodal rainfall pattern, lower livestock density and predominantly permeable soils i.e. soils of poor water holding capacity. Previous studies have shown that the impermeable soils, persistent heavy rainfall and high livestock density are associated with RVF occurrence [60, 76–80]. Interestingly, Beck and Sieber [81] have shown that the impermeable soils are among the soil type associated with high suitability for animal husbandry. The increased amount of precipitation in locations with impermeable soils is likely to provide suitable habitat for mosquito breeding and survival, and long term availability of pastures and water for livestock keeping compared with locations with permeable soils. Permeable soils are characterized by high proportions of sandy texture [82], and are therefore less likely to favour water stagnation over extended period of time. In contrast, impermeable soils are characterized by high proportions of clay and loamy texture which do not easily allow water to filter through resulting in periodic water stagnation and flooding during periods of prolonged rainfall [82]. Such flooding then leads to the hatching of RVFV infected Aedes mosquito eggs, which are considered to be the reservoirs and primary transmitters of the RVFV [78]. Colonization of the flooded areas by secondary vectors including Culex, Anopheles and Mansonia mosquitoes contribute to further virus transmission and spread between animals and humans [83]. The odds of RVF outbreaks have been shown to be more than eight times higher in locations with impermeable soils than locations with permeable soils [16].

Our model shows further that the highest probability of RVF occurrence occurs at the precipitation of the wettest quarter between 375 and 425 mm. This observation confirms the findings of a recent study in Tanzania that has shown that RVF outbreaks were associated with cumulative amount of rainfall > 400 mm during the previous two months [16]. These observations demonstrate the fundamental role of rainfall in the occurrence of RVF. Precipitation of the wettest quarter has been shown to be the proxy-attribute regulating habitat suitability for several RVF vectors including Aedes, Culex and Anopheles mosquitoes [31, 84–86]. Although distances to forest and wild animal protected areas were dropped out during the model building process, they have been reported to influence transmission dynamics of RVF. Forest has been reported to support breeding and survival of mosquitoes [87, 88]. Furthermore, wild terrestrial small mammals living in the forest have been reported to play role in the maintenance of RVFV [89]. The animal-mosquito cycling may involve low-level of infections in wild animals and these animals are likely to remain the reservoirs [78]. These observations suggest that animals and humans residing in or close to forest and wild animal protected areas are more likely to suffer from RVF than their counterparts.

The habitat suitability estimates of RVF occurrence presented in this study show a very high degree of visual agreement with the spatial distribution of RVF outbreaks in Tanzania [16]. Most of areas deemed to be the most suitable for RVF occurrence coincide with those that have reported RVF outbreaks in the past. Almost half (48.5%, n = 66) of the districts in the areas with higher suitability values had reported RVF outbreak in the past compared with 11/41 (26.8%) and 1/52 (1.9%) districts in the areas considered moderately and least suitable for RVF occurrence. A recent study reporting on the potential distribution of vectors responsible for RVF in Tanzania [32] provides reasonable visual agreement (by looking at the maps) with our model of habitat suitability for RVF occurrence suggesting, not surprisingly, that RVF suitable habitat is linked to vector distribution. However, the data on the distribution of potential vectors for RVF in Tanzania is very limited. Generation of more data on mosquito distribution in the country would be valuable input to improve the habitat suitability map. Furthermore, the ground-truthing for our model demonstrated that the odds of RVFV seropositivity corresponded with predicted suitability values.

It is worth noting that disease mapping is often limited by the resolution of the data available. Due to the small sample size of presence data points used to construct the ecological niche model and risk map in this study, it would be unrealistic to expect this model to describe fully the niche of RVF occurrence in the study area. Our predictive map shows that most RVF outbreaks have been reported in the areas predicted to be most suitable. However, we cannot exclude the possibility of under-reporting and/or sampling/reporting bias resulting particularly from the fact that most of past surveillance efforts have been conducted in the locations with known history of RVF occurrence, mainly in the northern Tanzania. This may have affected the predictive performance as the model was developed using existing presence only records of RVF outbreaks. The generated country habitat suitability map should be interpreted cautiously because most of the predictor variables included in the final model i.e. livestock density, rainfall pattern and soils were available at regional resolutions, and this may have contributed to the observed pattern suggesting that changes in the predicted probability of RVF occurrence largely coincide with regional boundaries. It is possible that the spatial distribution of some predictor variables considered in this study may have changed over time. For instance, the most current data for livestock density in Tanzania was available from the last national sample census of agriculture that was conducted in 2007/2008. Availability of most current data might have improved the predictive performance of our model.

In addition, the method by which background data are generated may not always provide the appropriate contrasts necessary for rigorous model calibration [90]. Background data can be generated in a number of different ways including random sampling and two-step strategies in which the species ecological niche is first defined using a profile method, and then data points are randomly generated within this constrained area. However, these different strategies can result in varying predicted spatial distribution of the species. Hanberry and others [91] found that while two-step strategies over-predicted species presence, due to too much environmental distance between the presence and ‘absence’ data, models based on random absences under-predicted species presence, due to too little environmental distance between the presence and absence points. However, based on trials using a simulated species, Wisz and Guisan [92] argue that, although randomly selected pseudoabsence data yield models with lower fit to the training data, they generally outperform models based on psedoabsences selected using a two-step method. They therefore suggest that randomly selected pseudoabsence data may be a reasonable alternative when real absences are unavailable [92].

Although we assumed that locations with higher proportions of RVFV-seropositive animals represented higher levels of habitat suitability for RVFV activity it is however not well known if animals that recover from natural RVFV infections are protected from development of clinical disease should RVF outbreaks occur in the future at a given location. Furthermore, the effect of herd immunity when a large percentage of animals have recovered with immunity from natural RVFV infection is not clearly known. Specific studies are needed to test these hypotheses.

It is also probable that, the inclusion of other potential predictor variables which were not available for consideration into our model, such as animal movement networks and distribution of vectors in the country, would have also improved the predictive performance. However, as this model describes the habitat suitability for RVFV activity, it is still of value. It is of note that our model predicted areas of suitable habitat in the western and south-eastern areas of the country that have never reported RVF outbreaks.

### Conclusion

The ecological niche modelling implemented in this study illustrates the extent of suitable habitat for RVF occurrence in Tanzania. The results suggest that the northern and central-eastern Tanzania have a higher probability of RVF occurrence than the rest of the country. Our model predicted areas of suitable habitat, the western and south-eastern areas of the country, beyond the known localities of RVFV activity. The modelled most suitable habitat for RVF occurrence in this study is characterized by high livestock density, moderate precipitation in the wettest quarter, predominantly impermeable soils and bimodal rainfall pattern. The findings of this study provide scientific evidence that can inform the design of cost-effective RVF prevention and control programmes targeting the identified high risk locations.
